# Superlithiophilic Amorphous SiO_2_–TiO_2_ Distributed into Porous Carbon Skeleton Enabling Uniform Lithium Deposition for Stable Lithium Metal Batteries

**DOI:** 10.1002/advs.201900943

**Published:** 2019-07-22

**Authors:** Pan Xue, Chuang Sun, Hongpeng Li, Jiajie Liang, Chao Lai

**Affiliations:** ^1^ School of Chemistry and Materials Science Jiangsu Normal University Xuzhou Jiangsu 221116 P. R. China; ^2^ School of Materials Science and Engineering National Institute for Advanced Materials Nankai University Tianjin 300350 P. R. China; ^3^ Tianjin Key Laboratory of Metal and Molecule‐Based Material Chemistry and Collaborative Innovation Center of Chemical Science and Engineering (Tianjin) Nankai University Tianjin 300350 P. R. China

**Keywords:** lithiophilic hosts, lithium metal anodes, lithium metal batteries, silica, titanium dioxide

## Abstract

Lithium (Li) metal anodes have garnered increasing interest in recent years as its high theoretical capacity and low electrochemical potential promises a myriad of opportunities for various applications. However, one critical issue to overcome is the inhomogeneous deposition of Li^+^ during the plating and stripping process. This inhomogeneous deposition could result in uncontrollable dendrite growth, further leading to poor coulombic efficiency, shorter lifecycles, and safety concerns due to internal short circuit and thermal runaways. To address these issues, a 3D porous core–shell fiber scaffold is presented, comprising of well‐dispersed SiO_2_, TiO_2_, and carbon, as superlithiophilic host materials for lithium anodes. The amorphous SiO_2_ and TiO_2_ allow for controllable nucleation and deposition of metal Li inside the porous core–shell fiber even at ultrahigh current densities of 10 mA cm^−2^. In addition, the interconnected conductive fiber with high porosity enables good electrical conductivity with fast ion transport and excellent mechanical strength to withstand massive Li loading during repeated cycles of stripping and plating. As a result, excellent cycling performance and high rate capability are observed in both symmetric cells and full cells, highlighting the feasibility of the proposed Li anode composite.

## 1 Introduction

In the push for green energy, rechargeable high‐energy batteries have garnered increasing attention in recent years.^1^ Among various candidates for these high energy batteries, metallic lithium‐based batteries have stood at the forefront due in part to the performance of high‐energy lithium–sulfur and lithium–oxygen batteries.^2^, ^3^, ^4^, ^5^ These batteries are capable of delivering a specific capacity ten times higher than commercial graphite anodes ubiquitously found in lithium‐ion batteries and demonstrate low electrochemical potentials.^2^, ^3^, ^4^, ^5^ However, in spite of these remarkable performance metrics, there are intrinsic limitations to these batteries. The inhomogeneous deposition of Li^+^, especially under high current density, can result in uncontrolled dendrite growth, which can penetrate the separator, leading to internal short circuit, thermal runaways, and even catastrophic cell failure. In addition, fresh Li dendrites can increase side reactions between lithium and organic electrolytes, resulting in poor coulombic efficiency.

Various strategies have been employed to address these issues and can generally be classified into two categories: interface regulation and structural design.^2^, ^3^, ^4^, ^5^ With respect to the former category, lithium nitrate, fluoroethylene carbonate, ionic liquids, Cs^+^, cationic surfactants, and so on, have been investigated to induce the formation of stable solid‐electrolyte interphase (SEI) layers or to control Li^+^ deposition at the anode‐electrolyte interface.^2^, ^6^, ^7^, ^8^, ^9^, ^10^, ^11^ However, even with the aid of these interfacial layers, uniform deposition is difficult as it is dependent on a plethora of external factors including ion diffusion, screw dislocation of atoms, and the morphology of the electrode surface.^2^, ^12^, ^13^ Regarding the latter category, various structural engineering techniques have been proposed, including processes such as fabrication of 3D carbon fiber‐based skeletons.^14^, ^15^, ^16^, ^17^, ^18^, ^19^, ^20^, ^21^, ^22^, ^23^, ^24^, ^25^ This specific technique offers several advantages:^26^ 1) the porous structure reduces the local current density and ensures sufficient Li ion flux; 2) the porous 3D skeleton accommodates the volumetric change of the Li anode during the plating and stripping progress; 3) Li is deposited on the interior of the 3D matrices, but not directly on the surface of the electrode, thus prohibiting dendrite growth. These aforementioned features are conducive to the production of a stable and dendrite‐free lithium anode.

Unfortunately, these attributes are predominately relegated to the initial lifetime of the device. Under continual use, the gradual formation of Li dendrite on the surface of these 3D matrix is inevitable since Li exhibits poor wettability on carbon host.^20^ This intrinsic low affinity between carbon and Li would lead to inhomogeneous Li^+^ flux distribution and generally bring about uneven Li nucleation and deposition especially under high current density, which in turn could result in the low coulombic efficiency and questionable safety as that using bare Li anodes.^17^, ^20^ Thus, the problem statement is to understand how to precisely control the deposition of Li onto the matrix. In recent years, materials such as silver nanoparticles, Al_2_O_3_, SiO_2_, MnO_2_, and ZnO have been investigated for use as lithiophilic materials to aid in the controlled deposition of Li, as nucleation and deposition of Li preferentially happen on the surface of polar metal or metal oxides.^27^, ^28^, ^29^, ^30^, ^31^, ^32^, ^33^ As such, the stronger the lithiophilic interaction, the better the electrochemical performance of Li composite anodes. Meanwhile, it should be emphasized that the ultrauniform distribution of such lithiophilic additives in highly conductive matrix is also necessary to avoid the aggregation of metal Li in the 3D scaffolds. However, the development of novel 3D conductive composite scaffolds containing well‐dispersed superlithiophlic additives is still in the early stage, and a facile and mild method to construct such host for Li‐metal anode is highly desirable to enable practical applications for Li‐metal batteries.

Herein, instead of conventional carbon‐based host, we propose a novel design of superlithiophlic, conductive, and 3D porous core–shell carbon fiber (henceforth denoted as PCSF) with amorphous SiO_2_ and TiO_2_ hybrid well distributed into the carbon skeleton. This unique host for Li‐metal anode is synthesized through a facile method including sequent steps of electrospinning, hydrolysis, polycondensation, and carbonization of the precursor mixture of tetraethyl orthosilicate, tetrabutyl titanate, and polyvinyl pyrrolidone. As confirmed by the computational results, the amorphous SiO_2_ and TiO_2_ show excellent affinity with Li and thus can guide the uniform Li nucleation and deposition even under high current density of 10 mA cm^−2^, which allows for effective suppression of Li dendrite growth. Moreover, the unique 3D porous core–shell architecture in conjunction with good mechanical properties of the carbon skeleton enables this superlithiophlic host to support massive Li deposition without obvious electrode volume change. As a result, the composite anode employing this superlithiophilic 3D porous fiber as host can work smoothly over 350 cycles at 4 mA cm^−2^ with low overpotential of ≈35 mV in symmetric cells. When paired with a LiNi_0.5_Co_0.2_Mn_0.3_O_2_ (NCM) cathode, the full cell can exhibit excellent rate performance up to 20 C (discharge ≈80 mAh g^−1^) and stable cycle performance up to 200 cycles at 10 C with a capacity decay of only 0.077% per cycle.

## 2 Results and Discussion

**Figure 1:**
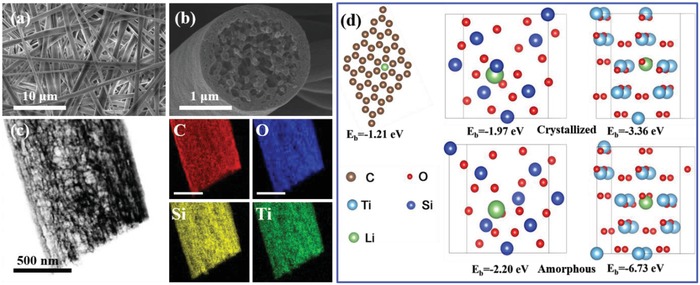
a) Top‐view SEM images of the top surface of the PCSF. b) Cross‐sectional SEM images showing PCSF hosts with a fiber diameter of 1–2 µm. c) Element mapping analysis of PCSF composite. d) Optimized geometrical structures and corresponding binding energies of a Li atom adsorbed on carbon, SiO_2_ (101) surface, TiO_2_ (101) surface, amorphous SiO_2_, and amorphous TiO_2_.

To fabricate the PCSF, uniform fibers were first obtained through electrospinning. Briefly, tetraethyl orthosilicate (TEOS), tetrabutyl titanate (TNB), and polyvinyl pyrrolidone (PVP) were dissolved in ethanol to obtain a homogeneous solution. The solution was subsequently electrospun, resulting in phase separation, hydrolysis, and polycondensation of TEOS and TNB. The hydrolysis and polycondensation process generates cross‐linked SiO_2_ and titanate, ultimately forming the desired 3D fiber‐based skeleton.^34^ The desired conductive 3D porous core–shell fiber was obtained via carbonization, with the morphology of the structure characterized by scanning electron microscopy (SEM) images depicted in **Figure** **1**a,b. These SEM images illustrate a 3D interconnected structure with electrospun fibers 1–2 µm in diameter. To further characterize the microstructure of the PCSF composite, transmission electron microscopy (TEM) was conducted, as seen in Figure S1 in the Supporting Information. The high‐resolution transmission electron microscopy (HRTEM) images reveal an amorphous structure with an absence of lattice fringes (Figure S1, Supporting Information), which is further confirmed by the X‐ray diffraction (XRD) spectrum in Figure S2 in the Supporting Information. Additional numerical characterization of the 3D porous core–shell structure is revealed through Brunauer–Emmett–Teller (BET) analysis and nitrogen adsorption/desorption isotherms. The BET surface area of PCSF is 151.7 m^2^ g^−1^, which provides a large contact area between the lithiophilic matrices and Li^+^. Furthermore, a typical IV hysteresis loop is observed, corresponding to a predominant pore size of 3.9 nm (Figure S3, Supporting Information).

The dispersion of the different materials is critical to the ultimate PCSF performance, as it enables smooth fibers to be generated during the Li plating process. To confirm the dispersion of the materials, energy dispersive spectrometry (EDS) was used to quantify the elemental mapping within the PCSF. The resultant figures are illustrated in Figure 1c, where it can be observed that Si, C, Ti, and O are all uniformly distributed on the fiber. And the contents of different elements in the composite are given in Table S1 (Supporting Information), for which the mass ratio of carbon and oxides is 26.6% and 73.4%, respectively. As a further confirmation of the elemental composition, X‐ray photoelectron spectroscopy (XPS) was also employed to analyze the PCSF composite (Figure S4, Supporting Information). Meanwhile, the core level spectra of Ti 2p and Si 2p are also shown in Figure S4b,c in the Supporting Information. After fitting, it can be concluded that Ti and Si elements mainly exist as TiO_2_ and SiO_2_ state as discussed above.^35^, ^36^, ^37^ This can be further confirmed by the Fourier transform infrared (FTIR) measurements (Figure S5, Supporting Information). As presented, the characteristic absorption bands for Ti—O—Ti bonds and Si—O—Si bonds can be detected,^38^, ^39^ confirming the coexistence of TiO_2_ and SiO_2_. Interestingly, Ti—O—Si bond is also observed,^40^ and this may result from the formation of small amount of titanium silicates during high temperature sintering. After broad elemental analysis, the relative content composition of the different components was analyzed through thermogravimetric analysis (TGA) (Figure S6, Supporting Information). The TGA measurement indicates that the lithiophilic metal oxides is ≈77.0%, of which SiO_2_ and TiO_2_ constitute 62.1% and 14.9%, respectively, calculated from the adding amount of tetraethyl silicate and tetrabutyl titanate. Although the content of carbon is not high (23 wt%), the carbon skeleton of PCSF still enables good electrical conductivity (2.16 × 10^−2^ S m^−1^), which ensures the rapid transport of electrons throughout the entirety of the scaffolds.

**Figure 2:**
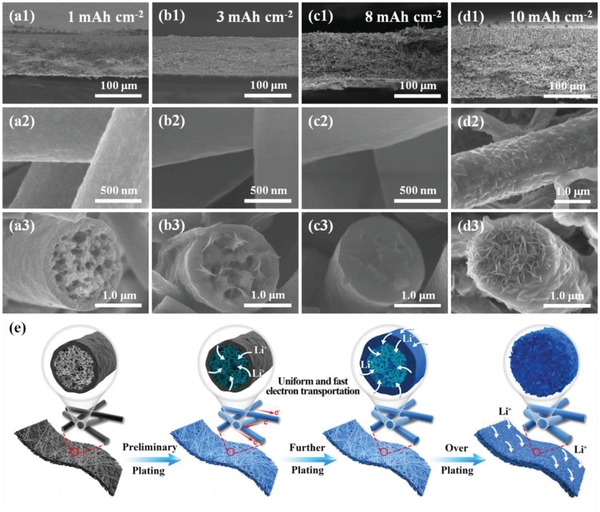
Cross‐sectional SEM images of the PCSF host after plating a1–3) 1 mAh cm^−2^, b1–3) 3 mAh cm^−2^, c1–3) 8 mAh cm^−2^, and d1–3) 10 mAh cm^−2^. e) Schematic illustration of the Li plating in the PCSF host at different states.

To clearly illustrate the roles of amorphous SiO_2_ and TiO_2_, density functional theory (DFT) calculations are conducted, and the results are presented in Figure 1d. It can be observed that while the binding energy of carbon and Li is only 1.21 eV, it can be enhanced to −3.66 and −1.97 eV for crystallized TiO_2_ and SiO_2_, respectively. For the amorphous TiO_2_ and SiO_2_ employed in this work, the binding energy can reach up to −6.73 and −2.20 eV, respectively, providing an even stronger anchoring effect for the adsorption, nucleation, and deposition of Li ions to control the plating behavior of Li.^2^, ^29^, ^30^, ^41^, ^42^ The superlithiophilic feature of amorphous oxides can well regulate the Li deposition inside the porous core–shell fiber, and this can be confirmed by Li plating experiments. **Figure** **2** illustrates SEM images of the Li@PCSF anode with different plating capacities conducted at a current density of 0.5 mA cm^−2^ in symmetric Li|Li cells. At 1 mAh cm^−2^ plating, a smooth surface and hollow structure are clearly observed. At a plating capacity of 3 mAh cm^−2^, most of the voids within the fiber have been filled (Figure 2b). When the plating capacity is increased to 8 mAh cm^−2^, the 3D porous core of the fiber is completely filled, though the surface of the PCSF remains smooth without any accumulation of Li on the surface (Figure 2c). To compare against the symmetric Li|Li cells, pure carbon fibers (CF) were prepared, and plated at 8 mAh cm^−2^. The resultant scaffolds can be observed in Figure S7 in the Supporting Information, where accumulation of Li inside the scaffolds can be clearly observed. These results suggest that the deposition of metal Li is well regulated by lithiophilic metal oxides up to a certain limit. When the plating capacity reaches 9 mAh cm^−2^ (Figure S8, Supporting Information), lithium begins to accumulate at the top, and the porous is completely filled. And after further increasing 10 mAh cm^−2^, more Li deposits can be observed on the surface of PCSF, and roughen the surface of the fiber (Figure 2d). This observation of surface accumulation indicates that the maximum platinum capacity of Li in the PCSF is ≈8 mAh cm^−2^. It is worthy to note that during increasing Li deposition, there is no enrichment of lithium metal at the intersection of the fibers (Figure 2a–c). This enables the PCSF to retain the loose 3D structure of the electrode, which is favorable for rapid transport of Li^+^, and can fundamentally eliminate the drastic volume change which occurs during the cycle. Taking the learnings of the aforementioned Li plating study, a schematic overview of the deposition behavior is presented in Figure 2e. During the deposition process, initial reduction of Li^+^ occurs first inside the 3D porous core of the fibers. Only after the inner volume of the fiber is filled will Li begin to deposit on the outer surface of the electrode.

The deposition of Li primarily on the inner surface of the hollow fiber is due largely in part to the strong anchoring effect of superlithiophilic SiO_2_ and TiO_2_ as illustrate in DFT calculations, which promotes nucleation and deposition of Li during the plating process. This anchoring effect of SiO_2_ and TiO_2_ were further verified by empirical fast plating experiments. Figure S9 in the Supporting Information shows SEM images of the PCSF after plating under different current densities at a fixed capacity of 3 mAh cm^−2^. It can be seen that even under ultrahigh current densities of 10 mA cm^−2^, the deposition of metallic lithium is still restricted within the hollow porous fiber. The preferring Li deposition inside the fiber mainly results from the different components of inner and outer fiber. As presented in Figure S10 in the Supporting Information, the inner fiber layer contains a higher content of lithiophilic SiO_2_ and TiO_2_ than that outer fiber layer, thus can well driving the Li preferential plating inside the fiber. Based on the aforementioned results, it can be concluded that the deposition of Li occurs preferentially on the surface of metal oxides, and that the hollow cavity and nanopores on the wall of the fiber provides the requisite space to store the deposited Li. This enables the fibers to provide a high loading of ≈8 mAh cm^−2^ without significant surface deposition on the electrode. At this loading (0.5 mA cm^−2^ for 16 h), the electrodes deliver a high reversible capacity of 1705.6 mAh g^−1^, which is ≈4.5 times higher than that of commercial graphite anodes, and sufficient to produce a high‐energy Li‐based battery (Figure S11, Supporting Information). Because surface deposition can be avoided even at high current densities, this design foreshadows that high cycling performance of PCSF based anodes can be expected for battery applications.

However, due to the high content of SiO_2_ and TiO_2_ in the PCSF, and the volume changes associated with the materials during Li plating, concerns remain regarding the structural stability of the nanoporous hollow fiber during the cycling process. To address this concern, Li plating of the PCSF was conducted at a current density of 0.5 mA cm^−2^ for 16 h (8 mAh cm^−2^), followed by stripping at the current density of 0.2 mA cm^−2^. After full extraction of Li, SEM measurements were conducted, with the results shown in Figure S12 in the Supporting Information. The SEM images reveal an intact, hollow, porous structure, with no cracks of the fiber observed. The morphology of the fiber confirms the structural stability of the PCSF during the Li plating and stripping process, which provides favorable conditions for long cycle life when the fiber is utilized as the host for metal Li.

**Figure 3:**
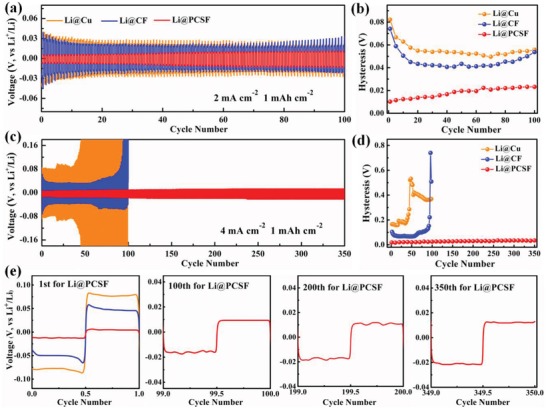
Cycling performance comparisons of symmetrical cells using a Li@Cu (orange), Li@CF (blue), and Li@PCSF composite anode (red) under a current density of a) 2 mA cm^−2^ and c) 4 mA cm^−2^ with a fixed capacity of 1 mAh cm^−2^. b,d) Comparison of voltage hysteresis of the Li plating/stripping with current densities of 2 and 4 mA cm^−2^. e) Detailed voltage profiles of the 1st, 100th, 200th, and 350th at current density of 4 mA cm^−2^, respectively.

Following all of the aforementioned characterization, it can be inferred that uniform deposition within the SiO_2_/TiO_2_/carbon‐based 3D porous core–shell fiber and retention of structural integrity during the plating and stripping of the Li are achievable in the PCSF design. By achieving these two integral functionalities, enhanced electrochemical performance can be realized. To demonstrate the improved electrochemical performance of the PCSF, symmetric cells were assembled with Li loading (Li@PCSF). The applied electrolyte comprised 1 m lithium bis(trifluoromethanesulfonyl)‐imide dissolved in dimethoxyethane (DME) and dioxolane (DOL) (1:1, v/v) with 0.2 m lithium nitrate (LiNO_3_) additives. The Li@PCSF electrode was obtained after plating at a current density of 0.5 mA cm^−2^ for 16 h to obtain a Li loading of 8 mAh cm^−2^. **Figure** **3**a presents a comparison of the electrochemical plating and stripping behavior of Li@PCSF, Li@CF, and Li@Cu electrodes at 2 mA cm^−2^ with capacity of 1 mAh cm^−2^. Due in part to the avoidance of surface deposition and the retention of the hollow structure, the Li@PCSF composite anode exhibits excellent cycle stability with no fluctuation and a low overpotential of 23.2 mV after 100 cycles (Figure 3b). In comparison, the Li@Cu and Li@CF anodes, have an overpotential of 55.8 and 53.7 mV, respectively, after 100 cycles. When the current density is increased to 4 mA cm^−2^ (Figure 3c), similar voltage profiles without significant increases in hysteresis are obtained for the Li@PCSF electrode, with the electrode able to maintain stable cycling performance up to 350 cycles. In contrast, the other two reference cells have much larger hysteresis, with the overpotential suddenly rising after 45 and 90 cycles for Li@Cu and Li@CF electrodes, respectively. This hysteresis and overpotential trend indicate the abundant growth of lithium dendrites on the surface of the electrode. Polarization voltage can well reflected the kinetic performance of different electrodes during cycling, and thus the advantages using Li@PCSF electrode can be more clearly demonstrated through the overpotential changes shown in Figure 3d. At higher current densities (4 mA cm^−2^), the Li@PCSF electrodes show an increase in overpotential to 34.7 mV after 350 cycles. However, significant increases in overpotential can be observed for Li@Cu and Li@CF electrodes after the 1st cycle. Enlarged images of the 1st, 100th, 200th, and 350th cycles of the symmetric cells are presented in Figure 3e. In the 1st cycle, a sharp voltage drop to 78.1 and 38 mV is observed for Li@Cu and Li@CF electrodes, respectively, in the initial stages of Li plating, corresponding to the nucleation process. In contrast, the Li@PCSF electrode exhibits a significantly smaller voltage drop of 11.8 mV, indicating homogeneous nucleation in the PCSF host materials. Furthermore, the voltage profile is observed to remain stable upon cycling. These observations suggest that the lithiophilic PCSF host is effective in guiding the nucleation and deposition process of metal Li, thus ensuring the high cycling performance in symmetric cells, especially at high current densities.

In addition to the stable cycle performance, the homogeneous dendrite‐free growth of Li in the PCSF host provides a favorable environment to grow a stable SEI layer and reduce the continuous consumption of electrolyte during cycling. This phenomenon can be confirmed by electrochemical impendence spectroscopy (EIS) measurements and the resulting Nyquist plots after cycling taken from the different electrodes (Figure S13, Supporting Information). The SEI resistance (26.1 Ω) and charge transfer impedance (13.9 Ω) of the Li@PCSF electrodes are notably lower than those of the Li@Cu and Li@CF anodes, indicating the creation of a stable solid–liquid interface after cycling on the surface of PCSF scaffolds.^43^, ^44^ The stable SEI film can well prohibit the continuous consumption of electrolyte, and inhibit the growth of lithium dendrites during cycling, thus leading to excellent cycling stability even at high current densities.

**Figure 4:**
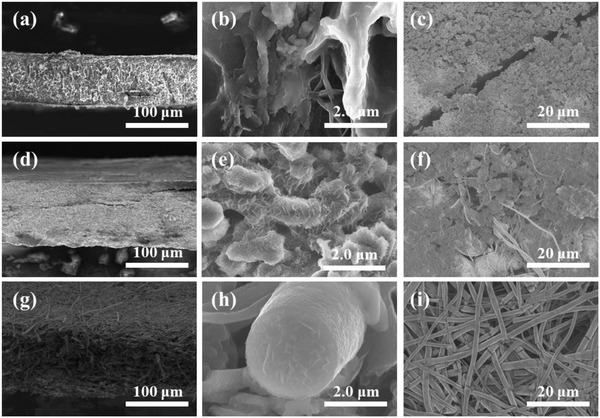
a–c) SEM images of Li@Cu, d–f) Li@CF, and g–i) Li@PCSF electrodes after 100 cycles at 4 mA cm^−2^ with capacity of 1 mAh cm^−2^.

To confirm the structural integrity of the electrodes after cycling tests, SEM images of different electrodes after 100 cycles were obtained and illustrated in **Figure** **4**. In all studies, the symmetric cells were tested in ether‐based electrolyte using an electrode preplating capacity of 8 mAh cm^−2^. The Li@Cu anodes show obvious cracks with massive mossy Li after 100 cycles of plating and stripping Li at current density of 4 mA cm^−2^. The Li@CF anodes show an intact 3D skeleton, but accumulated Li and dendritic Li formation both on the insides the scaffolds and on the surface of the electrode. Only the Li@PCSF electrode was observed to have a smooth surface with an absence of Li dendrites, validating the strong “anchoring” effect of the lithiophilic SiO_2_ and TiO_2_. The lack of dendrite formation is attributed to the uniform nucleation inside the fiber due to the lithiophilic SiO_2_ and TiO_2_.

**Figure 5:**
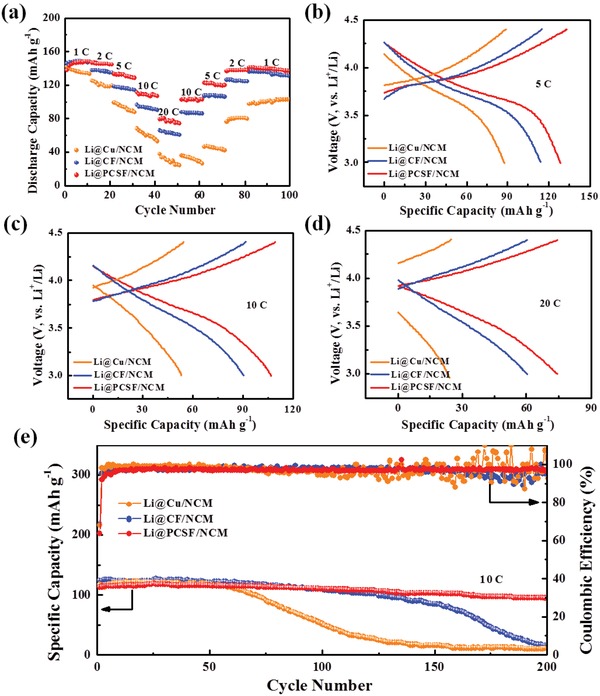
a) Cycling performance of Li@Cu/NCM, Li@C/NCM, and Li@PCSF/NCM full cells and b–d) corresponding voltage profiles at different rate. e) Long cycling performance of Li@Cu/NCM, Li@CF/NCM, and Li@PCSF/NCM full cells at 10 C (1 C = 155 mA g^−1^).

The Li@PCSF anode was incorporated in full cells after being paired with NCM cathodes. In these studies, all composite anodes were preplated at 8 mAh cm^−2^ and at a current density of 0.5 mA cm^−2^. **Figure** **5**a illustrates the cycle performance of the Li@PCSF/NCM Li@Cu/NCM and Li@CF/NCM full cells at rates ranging from 1 C to 20 C. The full cells with the Li@PCSF anode deliver an initial discharge capacity of 137.8 mAh g^−1^, lower than full cells utilizing Li@Cu (142.5 mAh g^−1^) and Li@CF anodes (146.2 mAh g^−1^). However, as the current density and number of cycles increase, the full cell with the Li@PCSF anode exhibits better cycle stability and rate capability. With increasing rates of 2 C, 5 C, 10 C, and 20 C, the discharge capacity is still well retained with values of 145.0, 128.6, 106.9, and 74.2 mAh g^−1^, respectively. In stark contrast, the Li@Cu and Li@CF anodes show a rapid decrease in the discharge capacity to 60.3 and 24.1 mAh g^−1^, respectively, at 20 C.

The discharge–charge profiles of different anodes are shown in Figure 5b–d. The potential profile is retained even at high rates of 20 C for the Li@PCSF anode. Furthermore, a significantly lower potential polarization upon cycling is observed when compared to the Li@Cu and Li@CF anodes, indicating the faster kinetics of the Li@PCSF anode. Figure 5e demonstrates the long‐term cycling performance of Li@PCSF/NCM, Li@Cu/NCM, and Li@CF/NCM full cells. The full cells with the Li@PCSF anode show an initial discharge of 114.4 mAh g^−1^, and stable cycle performance up to 200 cycles with an average capacity decay of only 0.077% per cycle. While both reference anodes also show higher initial capacity, rapid capacity decay is observed after 60 cycles for the Li@Cu anodes, and 130 cycles for the Li@CF anodes, respectively. After 200 cycles, the Li@Cu/NCM and Li@CF/NCM full cells are unable to further discharge and charge. In addition, the full cells demonstrate a steady high coulombic efficiency above 97.8% after initial several cycles.

The unique network structure and the “anchoring effect” of TiO_2_ and SiO_2_ on lithium ion deposition render PCSF an ideal host material for metal Li. The merits of this design can be summarized as follows: 1) a 3D intercommunicating skeleton can be obtained when PVP is carbonized at high temperatures, which enables rapid electron transfer and ion transport during lithium nucleation; 2) the uniform distribution of superlithiophilic TiO_2_ and SiO_2_ hybrid guides deposition of Li throughout the entire porous 3D core–shell fiber skeleton during ultrafast lithium plating and stripping, which prevents lithium from depositing on the surface of the electrode, and eliminates the formation of lithium dendrites; and 3) enhanced mechanical properties are obtained due to the physically interconnected electrospun fiber network, endowing the electrode with a strong resistance to volumetric expansion. As a result, this electrochemically stable and mechanically strong porous framework can support the forces caused by the deposition of large amounts of Li, thus maintaining a constant electrode size during the repeated plating and stripping of Li.^2^, ^26^

## 3 Conclusion

A simple, yet effective monitoring strategy to inhibit the growth of Li dendrites is presented by employing superlithiophilic 3D porous core–shell fiber scaffolds to construct high‐performance composite Li‐metal anodes. The deposition behavior of Li in the PCSF host materials was characterized and validated through both empirical and theoretical studies. Primary nucleation and deposition of metal Li inside the hollow SiO_2_/TiO_2_/carbon fiber is initiated by homogeneously dispersed metal oxides. This behavior allowed for dendrite‐free composite anodes, even at high current densities. Additionally, the interconnected conductive skeleton and hollow fibers enable fast ion and electron transport, high Li loading, and structural integrity of the matrix during the cycling process. As a result, flat voltage profiles and long‐term cycling stability were achieved even at high current densities. After being paired with NCM cathodes, the full cells demonstrated a high discharge capacity and remarkable cycling performance at 20 C.

## 4 Experimental Section

*Raw Materials*: 1 m lithium bis(trifluoromethanesulfonyl)‐imide and 0.2 m LiNO_3_ dissolved in DME and DOL (1:1, v/v), and 1 m lithium hexafluorophosphate and 0.1 m lithium bis(trifluoromethanesulfonyl)‐imide dissolved in a mixture of ethyl carbonate, dimethyl carbonate and ethyl methyl carbonate (1:1:1, v/v were obtained from dodochem Co., Ltd.). Li foils were obtained from Tianjin Zhongneng Lithium Industry Co., Ltd. Materials were stored and handled in an Mikrouna glove box under Ar atmosphere (<0.01 ppm H_2_O and <0.01 ppm O_2_). Cu and Al foils were purchased from Hefei Kejing Material Technology Co., Ltd. TNB (99%), PVP (*M* ≈ 1.3 m), and TEOS (99%) were purchased from Shanghai Aladdin Biochemical Technology Co., Ltd. Anhydrous ethanol (EtOH, 99%) and anhydrous acetate acid (HAc, 99%) were sourced from Sinopharm Chemical Reagent Co., Ltd.

*Preparation of 3D PCSF*: Fibers were prepared by electrospinning solutions of TNB/TEOS/HAc/EtOH/PVP following a method previously reported.^34^ First, PVP powder (2.0 g) was added to a mixture of EtOH/HAc (10 g/2.0 g) and stirred for 2 h. Following stirring, TNB (1.0 g) and TEOS (3.5 g) were added and stirred for 0.5 h until a homogeneous precursor solution was obtained. The precursor solution was injected into a syringe with a stainless steel spinneret (16 G), with a voltage of 20–23 kV applied to the spinneret. The distance between the flat receiver and stainless steel spinneret was 15 cm. The temperature and relative humidity were maintained at 45 °C and 35%, respectively. Fibers were collected in aluminum foil, dried overnight at 30 °C in air, and calcinated at 700 °C for 6 h at a heating rate of 5 °C min^−1^ in argon.

*Characterization*: Morphological and structural characterizations were conducted using a Hitachi SU‐8010 SEM. XRD spectra were obtained from a Bruker‐D8 ADVANCE X‐ray diffractometer. TEM images were taken using a transmission electron microscope JEM‐2800. XPS analysis was performed using an ESCALAB 250Xi system from Thermo Scientific. TGA studies were done using a TA Instruments Q50 in air at a heating rate of 10 °C min^−1^, with temperatures spanning room temperature to 750 °C. The nitrogen adsorption–desorption isotherms were demonstrated using a Quantachrome Autosorb‐IQ2 at 77 K. Specific surface areas were illustrated by Brunauer–Emmett–Teller analysis. The four‐probe conductivity test was measured by using a LTD ST‐2258C from Suzhou Lattice Electronics Co. FTIR spectrum was recorded with KBr pellets using a Bruker Optics Tensor 27 FTIR spectrophotometer.

*DFT Calculations*: DFT analysis was conducted using the Vienna Ab initio Simulation Package (VASP) code,^45^, ^46^ which was implemented under the projected augmented wave (PAW) method with the generalized gradient approximation of Perdew–Burke–Ernzerhof (PBE).^47^, ^48^ The plane wave basis was set to 350 eV. The convergence criteria for the total energies and forces of the electronic were set as 10^−5^ eV and 0.02 eV Å^−1^, respectively. Using molecular dynamics (MD) simulations, a thermal annealing was carried out to get the amorphous configuration of crystalline SiO_2_ and TiO_2_. The annealing simulation started from 300 K and ended at 970 K stepping in 10 K intervals. At each temperature, 10 ps NVT MD simulation was performed with a time step of 1 fs. Temperatures were controlled using the stochastic collision method proposed by Andersen. The binding energy (BE) of the Li atom in the crystal or amorphous configuration of SiO_2_ and TiO_2_ were calculated as follows

$$\mathrm{BE}=E_{\mathrm{total}}-E_{o}+E_{\mathrm{Li}}$$

where *E*_total_, *E*_o_, and *E*_Li_ were the total energy of one Li atom binding to the Carbon (C), SiO_2_, and TiO_2_, for each model Carbon (C), SiO_2_ and TiO_2_ without the binding of Li, and for one Li atom, respectively.

*Electrochemical Measurements*: For symmetric cell testing, the electrodes were assembled with symmetric cell configurations into coin cells. The as‐prepared 3D hollow Li@PCSF material with an area of 0.785 cm^2^ was used as the working electrode, while lithium foil was employed as the counter electrode. The area capacity of the working electrode was 8 mAh cm^−2^, and was prepared via lithium electrodeposition at a current density of 0.5 mA cm^−2^. The electrolyte used was 1 m lithium bis(trifluoromethanesulfonyl)‐imide and 0.2 m LiNO_3_ dissolved in DME and DOL (1:1, v/v). The cycling performance was tested at current densities of 2 and 4 mA cm^−2^. EIS were obtained using an electrochemical workstation (Solartron 1287) with an amplitude of 5 mV and a frequency range between 10 mHz and 100 kHz.

For the testing of Li@PCSF/NCM full cells, NCM electrodes were fabricated by mixing NCM, Super P, and binder polyvinylidene fluoride with a mass ratio of 7:2:1. The areal mass loading was 1.0 mg cm^−2^ for the cathode electrode. The Li@PCSF, Li@Cu, and Li@CF were employed as anodes with 8 mAh cm^−2^ lithium preplating. The electrolyte used in the full cells was 1 m lithium hexafluorophosphate and 0.1 m lithium bis(trifluoromethanesulfonyl)‐imide dissolved in a mixture of ethyl carbonate, dimethyl carbonate and ethyl methyl carbonate (1:1:1, v/v). The rate performance of the full cells was tested at various current densities ranging from 1 C to 20 C and then back to 1 C (1 C = 155 mA g^−1^). The cutoff potentials of charge and discharge were set at 4.4 and 3.0 V (vs Li^+^/Li).

## Conflict of Interest

The authors declare no conflict of interest.

## Supporting information

Supplementary
