# Supplementary material for: Superlithiophilic Amorphous SiO2–TiO2 Distributed into Porous Carbon Skeleton Enabling Uniform Lithium Deposition for Stable Lithium Metal Batteries
Source: Adv Sci (Weinh). 2019 Jul 22;6(18):1900943. doi: 10.1002/advs.201900943 (PMC6755548; doi:10.1002/advs.201900943)
Supplement: Supplementary file 1 — Supplementary [file ADVS-6-1900943-s001.pdf]

Copyright WILEY-VCH Verlag GmbH & Co. KGaA, 69469 Weinheim, Germany, 2019.

ADVANCED SCIENCE
Open Access

# Supporting Information

for *Adv. Sci.,* DOI: 10.1002/advs.201900943

# Superlithiophilic Amorphous $SiO_2$–$TiO_2$ Distributed into Porous Carbon Skeleton Enabling Uniform Lithium Deposition for Stable Lithium Metal Batteries

*Pan Xue, Chuang Sun, Hongpeng Li, Jiajie Liang,* and Chao Lai**

Copyright WILEY-VCH Verlag GmbH & Co. KGaA, 69469 Weinheim, Germany, 2019.

# Supporting Information

**Superlithiophilic Amorphous $SiO_2$-$TiO_2$ Distributed into Porous Carbon Skeleton Enabling Uniform Lithium Deposition for Stable Lithium Metal Batteries**

*Pan Xue, Chuang Sun, Hongpeng Li, Jiajie Liang* and Chao Lai**

Mr. P. Xue, Mr. C. Sun, Prof. C. Lai
School of Chemistry and Materials Science
Jiangsu Normal University
Xuzhou, Jiangsu 221116, P.R. China
E-mail: laichao@jsnu.edu.cn
Mr. P. Xue, Dr. H. P. Li, Prof. J. J. Liang
School of Materials Science and Engineering
National Institute for Advanced Materials
Nankai University
Tianjin 300350, P.R. China
E-mail: liang0909@nankai.edu.cn
Prof. J. J. Liang
Tianjin Key Laboratory of Metal and Molecule-Based Material Chemistry and Collaborative Innovation Center of Chemical Science and Engineering (Tianjin)
Nankai University
Tianjin 300350, P.R. China
Mr. P. Xue and Mr. C. Sun contributed equally to this work.

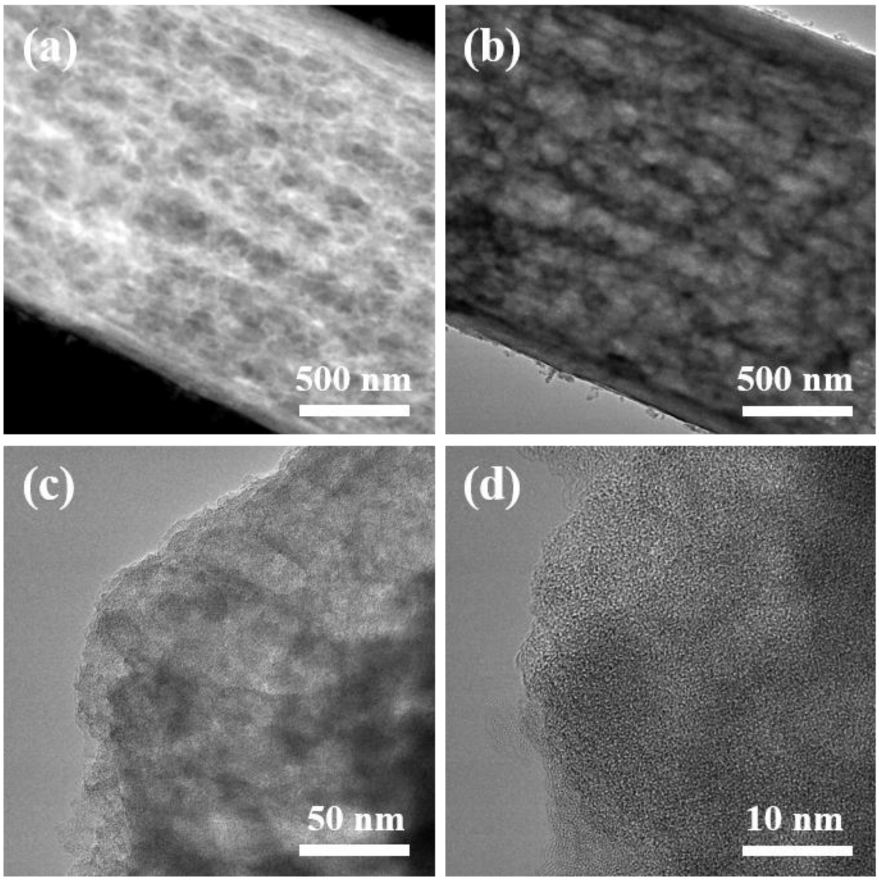


**Figure S1.** TEM (a,b) and HRTEM (c,d) images of the PCSF.

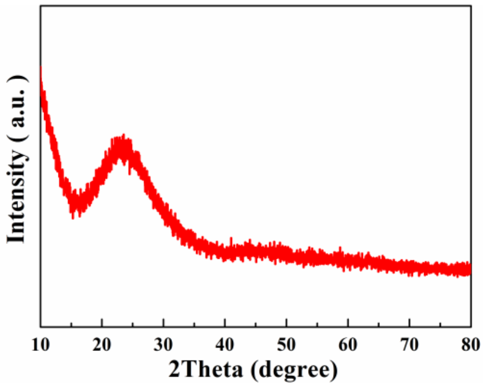


**Figure S2.** XRD patterns of the PCSF heat-treated at 700 $^{o}$C.

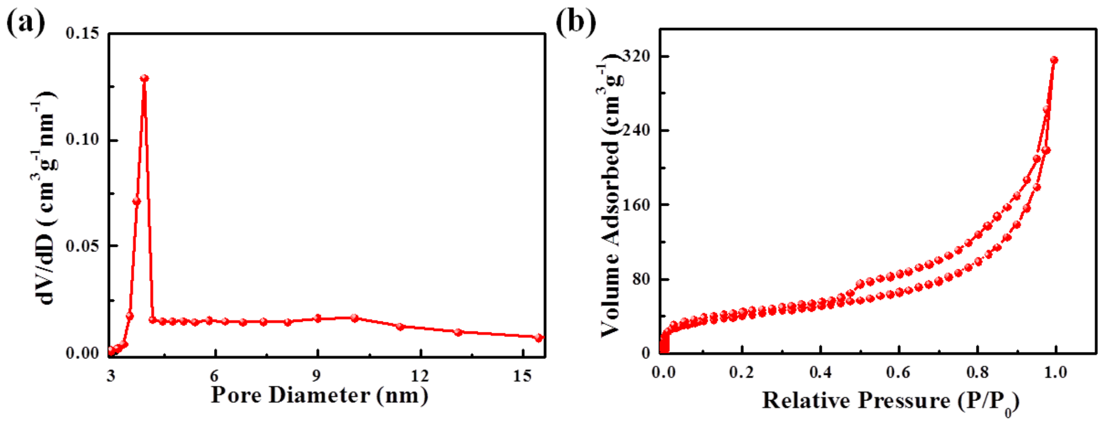


**Figure S3.** (a) Pore size distribution curves and (b) nitrogen adsorption-desorption isotherms of the PCSF.

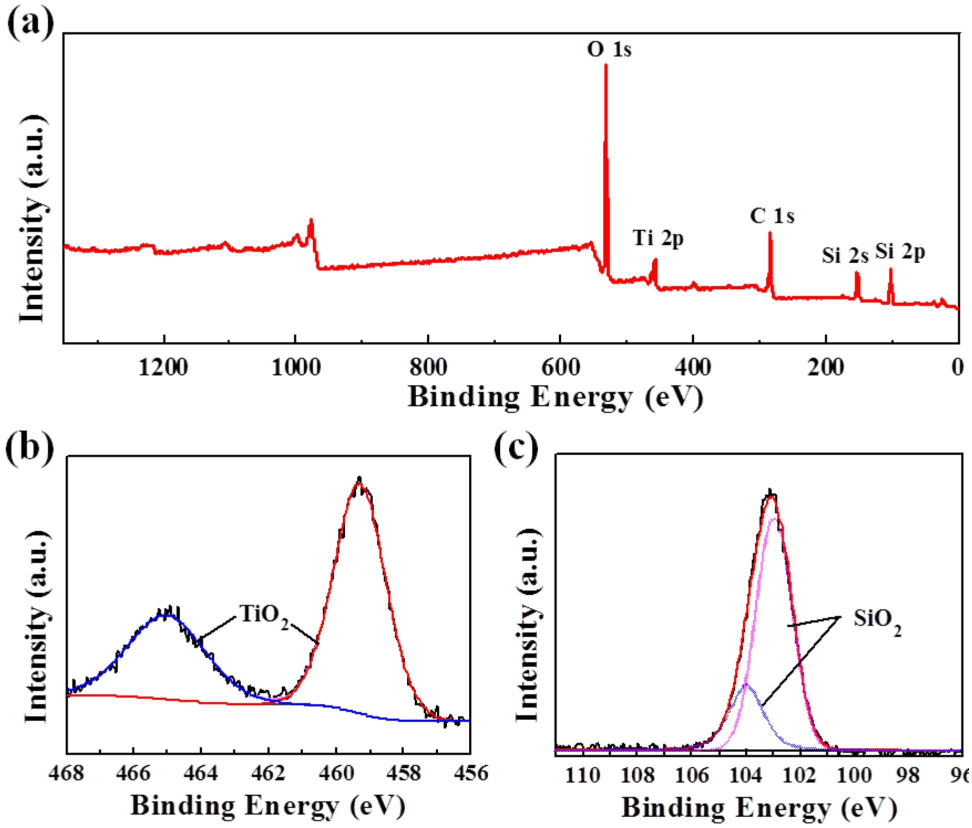


**Figure S4.** (a) XPS characterization of the PCSF composite, (b) core-level spectra of Ti 2p peaks, (c) core-level spectra of Si 2p peaks.

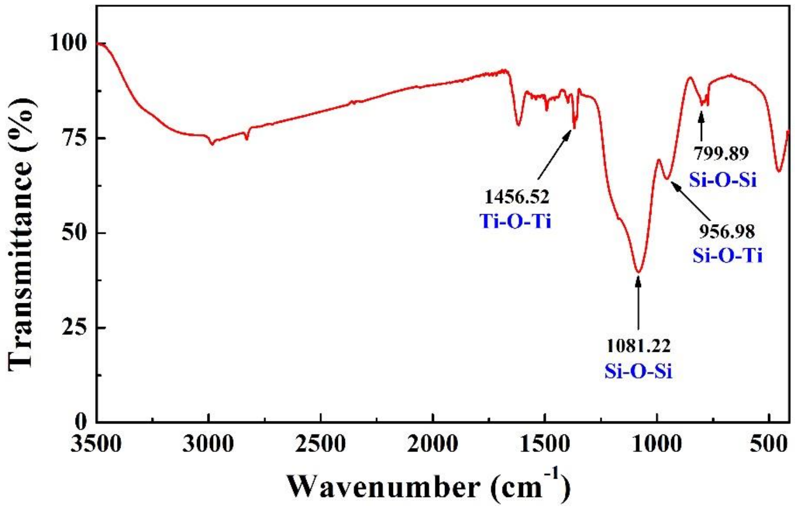


**Figure S5.** FTIR spectrum of PCSF composite.

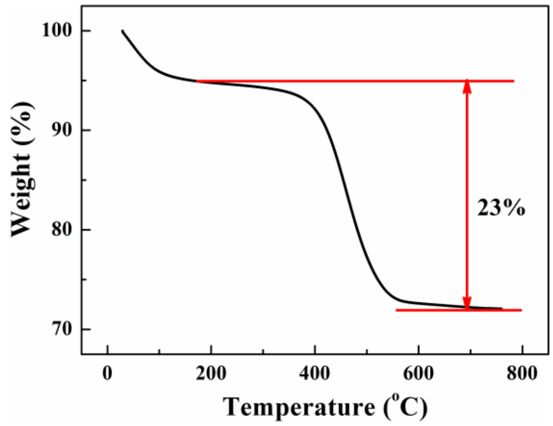


**Figure S6.** Thermogravimetric analysis (TGA) of the PCSF from room temperature to 750 $^{o}C$ at a heating rate of 10 $^{o}C$ $min^{-1}$ under air atmosphere.

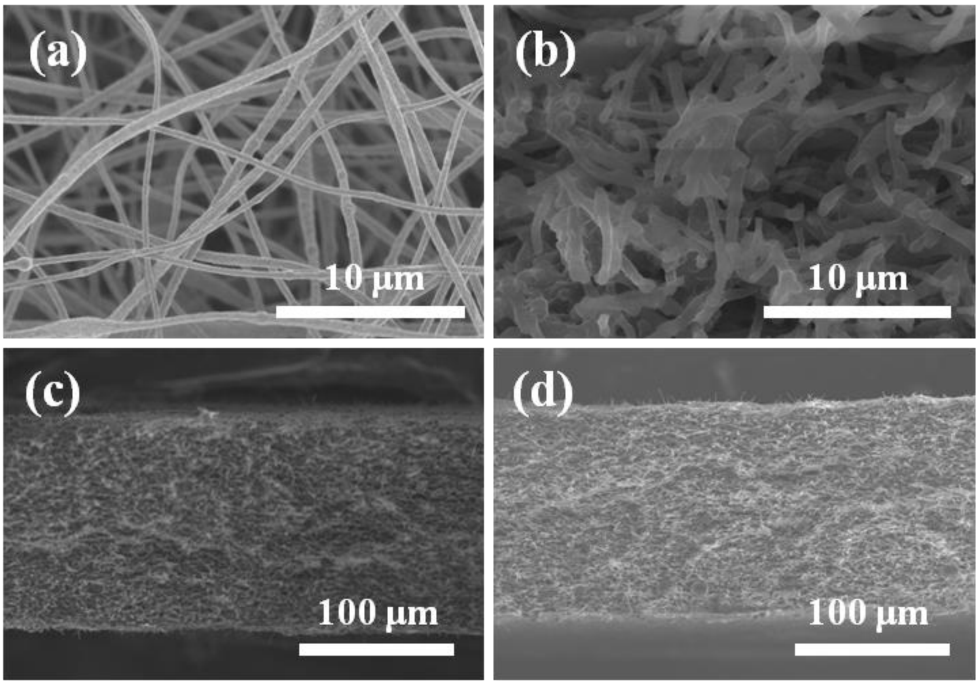


**Figure S7.** The SEM images of the CF (a, c) before depositing lithium metal and (b, d) after electrodeposition with high area capacity of 8 mAh $cm^{-2}$ lithium metal.

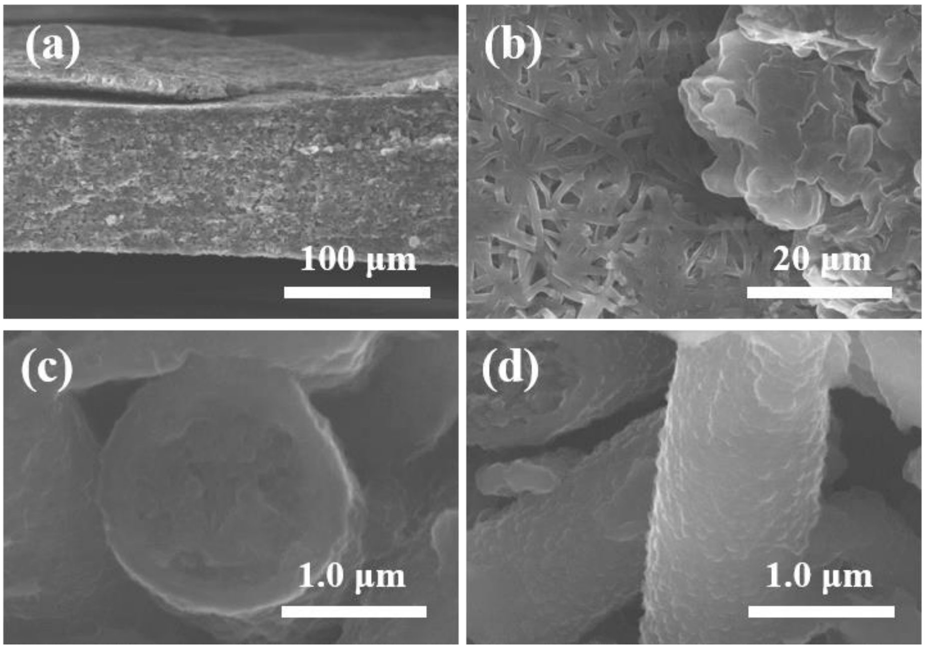


**Figure S8.** (a, c, d) Cross-sectional SEM images of PSCF host after Li plating of 9 mAh $cm^{-2}$, (b) Top-view SEM images of PCSF after Li plating of 9 mAh $cm^{-2}$.

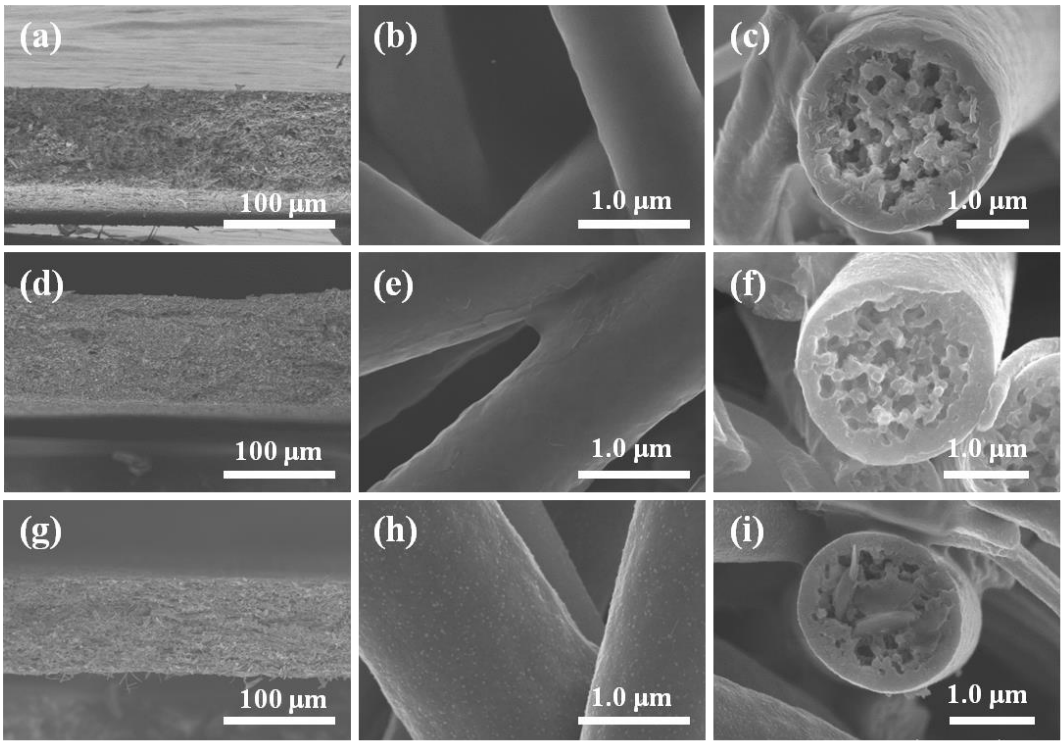


**Figure S9**. Cross-sectional SEM images of the PCSF host after plating 3 mAh cm$^{-2}$ under the different current densities (a-c) 2 mA cm$^{-2}$, (d-f) 5 mA cm$^{-2}$, (g-i) 10 mA cm$^{-2}$.

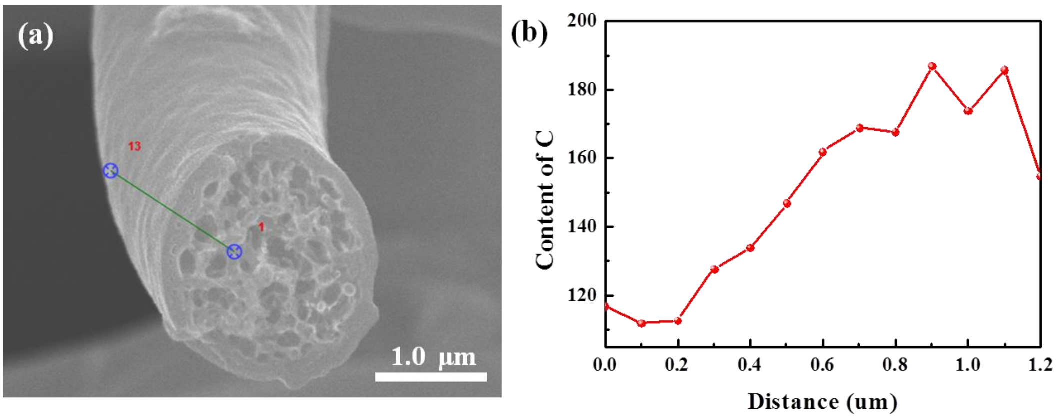


**Figure S10** EDX spectrum profile scanning data for the carbon atom of the PCSF.

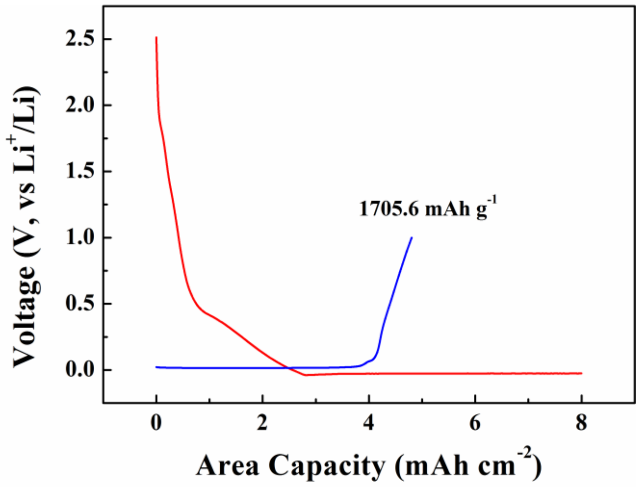


**Figure S11.** Voltage profile of initial plating 8 mAh cm$^{-2}$ and then stripping Li from the Li@PCSF to 1.0 V (vs. $Li^+$/Li).

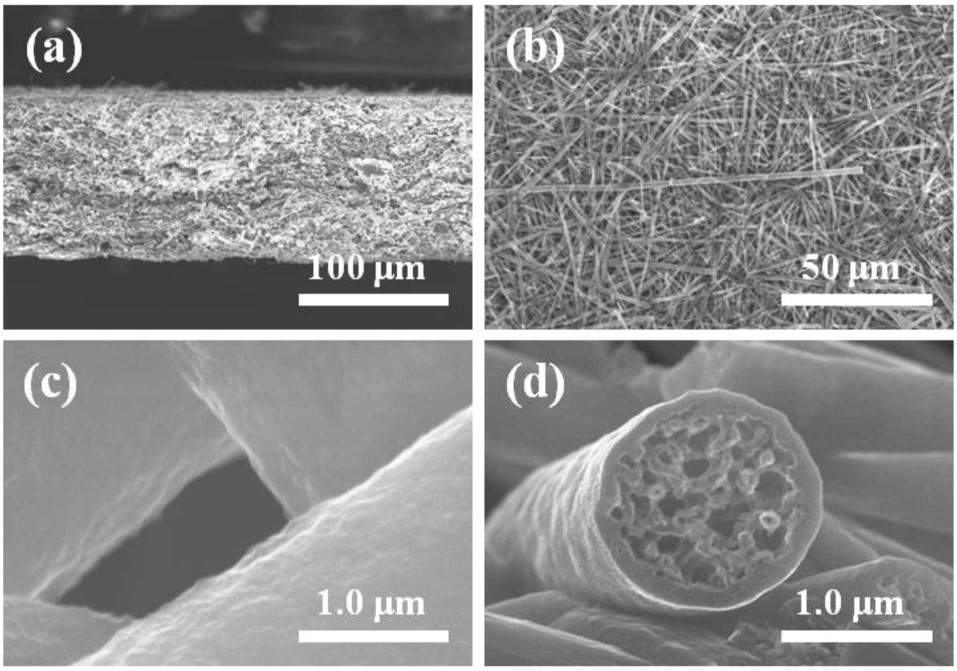


**Figure S12.** (a, c, d) Cross-sectional SEM images and (b) Top-view SEM images for the surface morphology of PCSF after plating at 0.5 mA cm$^{-2}$ and then stripping at the current density of 0.2 mA cm$^{-2}$.

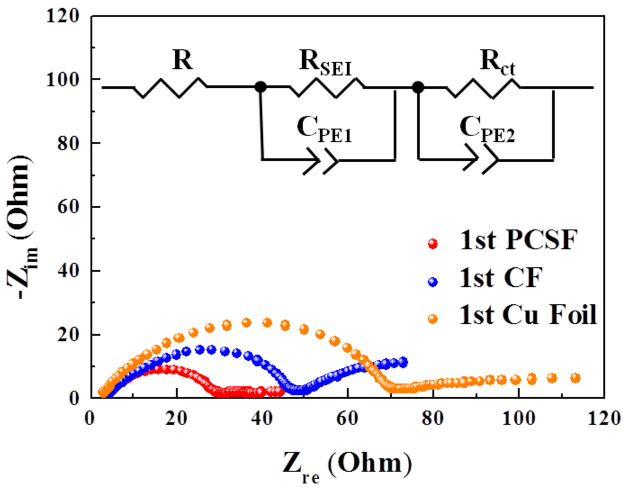


| Fitting results | | | | |
|---|---|---|---|---|
| | | PCSF | CF | Cu Foil |
| 1st cycle | $R_{SEI}$ （ohm） | 26.14 | 46.01 | 70.04 |
| | $R_{ct}$ （ohm） | 13.89 | 40.10 | 25.20 |

**Figure S13.** Impedance performance of Li@PCSF, Li@Cu and Li@CF composite anodes after 1 cycle of Li plating/stripping at 1 mAh cm$^{-2}$ at the current density of 2 mA cm$^{-2}$.

**Table S1** Quantitative analysis of elements C, O, Si and Ti in PCSF composite fiber.

| Element | Mass% | Atom% |
|---|---|---|
| C | 26.6 | 39.11 |
| O | 35.2 | 38.74 |
| Si | 31.2 | 19.56 |
| Ti | 7.0 | 2.58 |
